# Supplementary material for: AffyMAPSDetector: a software tool to characterize Affymetrix GeneChip™ expression arrays with respect to SNPs
Source: BMC Bioinformatics. 2007 Jul 30;8:276. doi: 10.1186/1471-2105-8-276 (PMC1959249; doi:10.1186/1471-2105-8-276)
Supplement: Additional file 13 — AffyMAPSDetector v1 source code. AffyMAPSDetector source code. [file 1471-2105-8-276-S13.zip › AffyMAPSDetector_v1_src/doc/affychip/snpdetector/util/PmMmHeatmapViewerModel.Row.html]

PmMmHeatmapViewerModel.Row


|  |  |  |  |
| --- | --- | --- | --- |
| **Overview** \| **Package** \| **Class** \| **Use** \| **Tree** \| **Deprecated** \| **Index** \| **Help** | | |  |
| **PREV CLASS**   **NEXT CLASS** | **FRAMES**    **NO FRAMES**     **All Classes** |  |  |
| SUMMARY: NESTED \| FIELD \| CONSTR \| METHOD | DETAIL: FIELD \| CONSTR \| METHOD |  |  |


---


## affychip.snpdetector.util Class PmMmHeatmapViewerModel.Row

```
java.lang.Object
  affychip.snpdetector.util.PmMmHeatmapViewerModel.Row
```

**All Implemented Interfaces:**: java.lang.Comparable

**Enclosing class:**: PmMmHeatmapViewerModel

---

public class **PmMmHeatmapViewerModel.Row** extends java.lang.Object implements java.lang.Comparable

Title: Probe SNP Detector

Description: It holds a row's data for a table. In addition each row
has a row-header and row-annotation, if any.

Copyright: Sunita Kumari -- Copyright (c) 2004

Company: George Mason University

**Version:**
:   1.1

**Author:**
:   Sunita Kumari

---

|  |  |
| --- | --- |
| **Method Summary** | |
| `int` | `compareTo(java.lang.Object that)` |
| `java.lang.String` | `toString()` |

**Methods inherited from class java.lang.Object**

`clone, equals, finalize, getClass, hashCode, notify, notifyAll, wait, wait, wait`

**Method Detail**

### compareTo

```
public int compareTo(java.lang.Object that)
```

:   **Specified by:**: `compareTo` in interface `java.lang.Comparable`

---


### toString

```
public java.lang.String toString()
```


---


|  |  |  |  |
| --- | --- | --- | --- |
| **Overview** \| **Package** \| **Class** \| **Use** \| **Tree** \| **Deprecated** \| **Index** \| **Help** | | |  |
| **PREV CLASS**   **NEXT CLASS** | **FRAMES**    **NO FRAMES**     **All Classes** |  |  |
| SUMMARY: NESTED \| FIELD \| CONSTR \| METHOD | DETAIL: FIELD \| CONSTR \| METHOD |  |  |


---
